# Endoscopic Repair of Gastrocolic and Colocutaneous Fistulas Complicating Percutaneous Endoscopic Gastrostomy Tube

**DOI:** 10.1155/2020/7262514

**Published:** 2020-02-11

**Authors:** Chukwunonso Chime, Ahmed Baiomi, Kishore Kumar, Harish Patel, Anil Dev, Jasbir Makker

**Affiliations:** Department of Gastroenterology, Bronx Care Health Systems–Affiliate of Mount Sinai Hospital Systems, Bronx, NY 10457, USA

## Abstract

Percutaneous endoscopic gastrostomy (PEG) tube feeding has become one of the options for supplemental feeding in a selected group of patients. It is a generally safe procedure usually undertaken by a gastroenterologist or a surgeon in most cases but with over 200,000 tubes being placed yearly, there is bound to be complications. Some of the encountered complications include bleeding, site infection, tube migration, and inadvertent creation of fistula. We present our index patient admitted from a long-term care facility for feculent vomiting and fecal material through the PEG tube. Imaging and colonoscopy confirmed the presence of both a gastrocolic and a colocutaneous fistula, both closed endoscopically with an over-the-scope and through-the-scope clips, respectively. Feeding through a nasogastric tube was resumed after 48 hours, and by the second week of admission, the patient was discharged back to the facility after placement of a new PEG tube.

## 1. Introduction

The use of a percutaneous endoscopic gastrostomy (PEG) tube for the maintenance of nutritional needs has gained increasing popularity since it was introduced in 1980s [1]. Compared to surgical gastrostomy tubes, PEG tubes have become the preferred method of gaining enteral access in patients that need feeding for a prolonged period [2]. There are still concerns for complications resulting from this route of enteral access. Complications, though rare, include bleeding, infection, inadvertent perforation of the bowel, and aspiration [3]. We present a case of an elderly male patient who had a PEG tube placed for long-term feeding, presented to our hospital with complaint of feculent vomiting and was noted to have a gastrocolic and a colocutaneous fistula, both repaired endoscopically.

## 2. Case Report

A 64-year-old male ventilator-dependent patient presented to the emergency department (ED) from a nursing home after he was noted with feculent emesis and fecal effluent from his gastrostomy tube. Two months earlier, the patient had a cardiac arrest in another hospital, resulting in anoxic brain injury. He required percutaneous endoscopic gastrostomy (PEG) tube insertion with tracheostomy prior to discharge to the nursing home. His other medical comorbidities were diabetes mellitus, chronic obstructive pulmonary disease, and right lower extremity deep vein thrombosis. In the ED, his vital signs were within normal limits. On physical examination, he was nonverbal and ventilator dependent through the tracheostomy tube with feculent material noted on PEG tube aspiration and rest of the examination was unremarkable. Laboratory investigations revealed hemoglobin of 9.1 g/dL, leukocyte count of 8.9 K cells/*µ*L with neutrophil predominance, and platelet count of 619 K cells/*µ*L.

**Figure 1:**
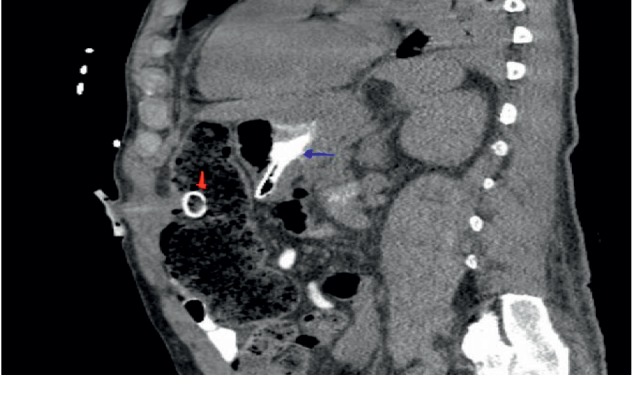
PEG tube in the transverse colon (red pointer) and contrast in the stomach (blue pointer) tracking through the fistula.

**Figure 2:**
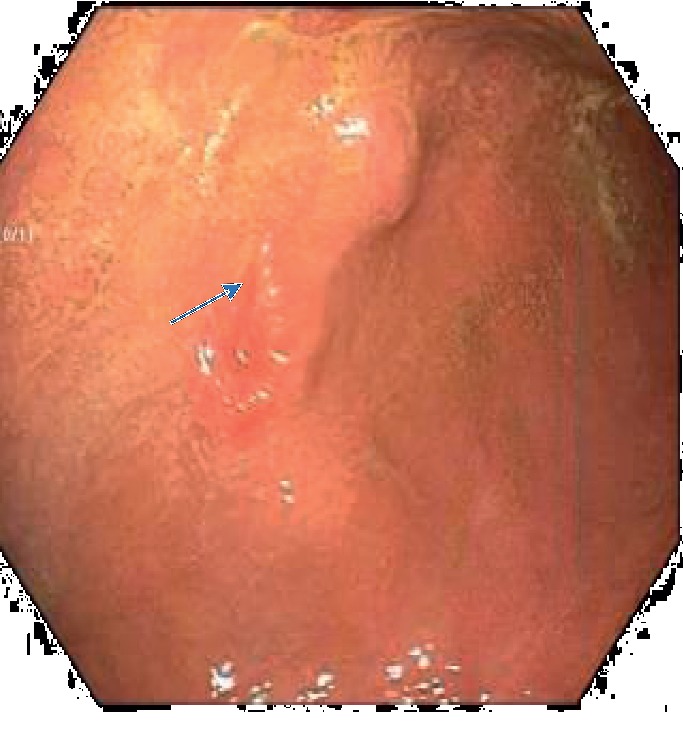
Gastrocolic fistula (blue arrow).

**Figure 3:**
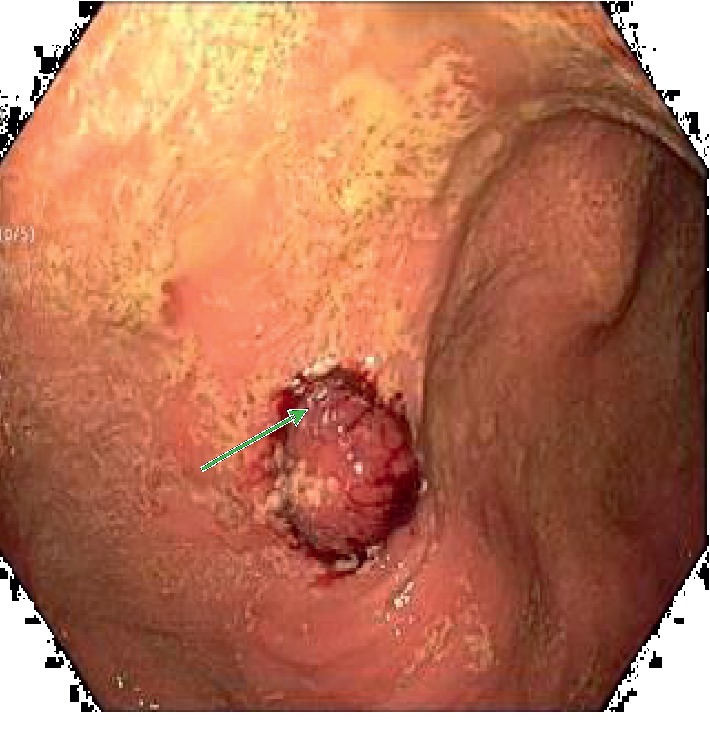
After over-the-scope repair (green arrow).

**Figure 4:**
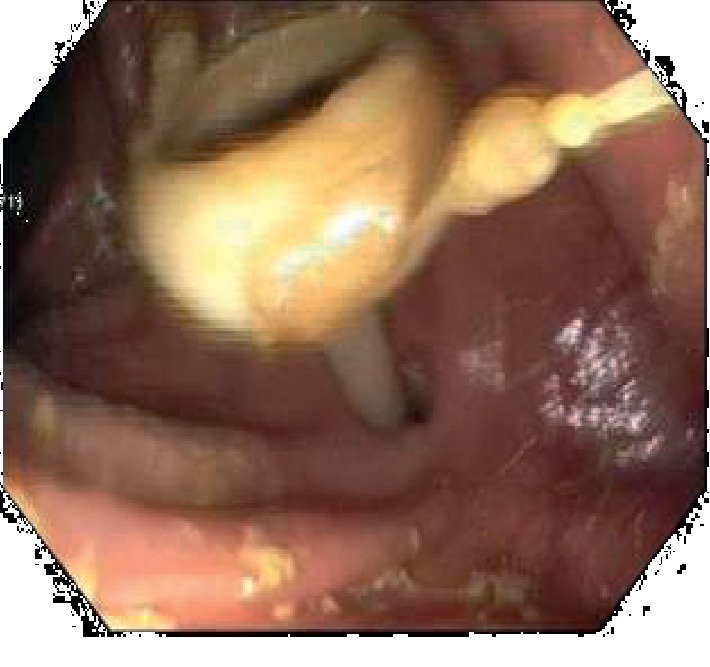
PEG tube internal bumper in the colon.

**Figure 5:**
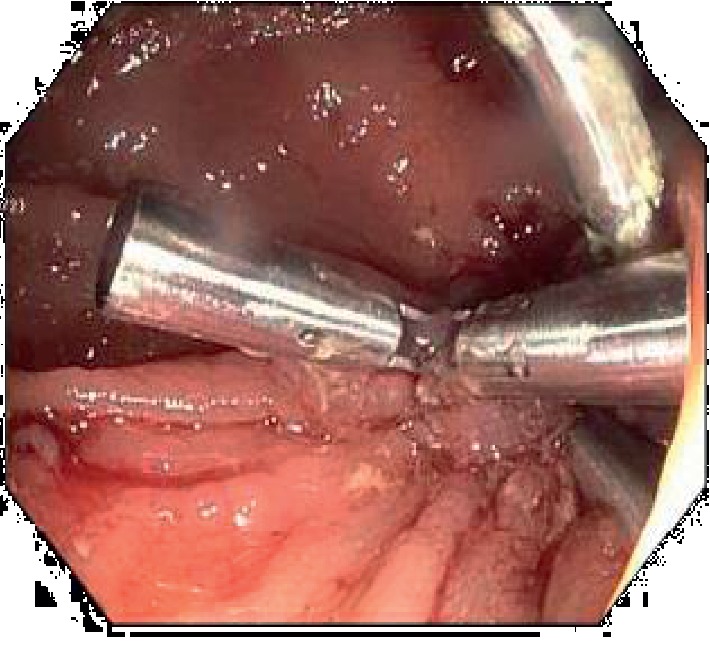
Repair of colocutaneous fistula.

He had a computed tomography (CT) scan done with oral contrast in the ED, and it showed a gastrostomy tube that appeared to be in the transverse colon (see Figure 1). On admission, he had an upper endoscopy showing feculent material in the stomach with evidence of a fistulous opening suspected to be in communication with the colon. The diameter was determined to be less than 10 mm, and this was repaired with an over-the-scope clip (OTSC) (see Figures 2 and 3). He also had a colonoscopy that revealed an internal bumper of the PEG tube located in the transverse colon, and this was cut externally at skin level and retrieved with a Roth net. The colocutaneous fistula could not be repaired with an OTSC as the location in the transverse colon could not be reached with a gastroscope, and this was subsequently repaired with through the scope clips using a pediatric colonoscope (see Figures 4 and 5). The success of the procedures was ascertained by adequate insufflation of the stomach and colon without air leak. Enteral feeding was resumed after 48 hours using a nasogastric tube access.

The patient was subsequently treated with antibiotics for a few days and had an unremarkable hospital course; 10 days after the repair of the fistulas, he had another PEG tube placed before being discharged back to the nursing home.

## 3. Discussion

Many patients with a spectrum of chronic medical illnesses and who are unable to meet their caloric needs through oral feeding require alternative means of achieving adequate nutrition through the enteral route. Nasogastric tubes can aid in creating access for nutrition with a drawback that they are short term, may cause irritation, bleeding, infection of nose, and throat, and may require the need to replace them when they dislodge. There was a need to create better long-term access, and PEG tubes have become a safe and effective means to achieve this need [4], with over 216,000 PEG tubes placed in the United States annually [5]. In general, there are two methods of PEG tube insertion and these utilize either the pull or push technique, resulting in the passage of a plastic tube through the skin to the stomach, anchored with an internal and external bumper. After placement of a PEG tube, a tract is usually formed from the skin, through subcutaneous tissue to the stomach. This tract begins to mature in 7 to 10 days, though in certain situations like immunosuppression or malnourishment, it may take longer [3].

Complications can be evident in the first few days due to lack of maturity of the PEG tract [1]. The incidence rate for major complications in adults after PEG tube placement has been reported to be 3% in one of the series [6]. These complications include bleeding, PEG site infection, dislodgement, aspiration, bowel trauma, and fistulous tracts [3, 7]. One of the major complications that could be encountered after PEG tube placement is formation of a false tract, such as a gastrocolic and/or colocutaneous fistula [8], as was the case with our patient. This is usually thought to result from juxtaposition of the colon between the stomach and the skin during the PEG insertion, resulting in a feeding tube passing through the colon, usually the transverse colon, before being anchored in the stomach [9]. Adequate air insufflation of the stomach can help prevent this complication by displacing the colon away from the path of the PEG tube [5]. Also, a two-step process that involves transillumination and finger indentation can help verify proper apposition of the abdominal wall with the gastric wall [10].

Suspicion for dislodged PEG with a resulting fistula should arise in patients with feculent vomiting or feculent material through the PEG as was the case in our patient. Recurrent postprandial diarrhea, though not present in our case, can also occur when the PEG tube is in the colon, resulting from direct feeding of the isotonic formula and has been described in literature [9]. Various modalities have been used to diagnose and delineate suspected misplacements of a PEG tube. A tubogram is deemed the test of choice as compared to colonoscopy or other imaging studies [11]. Caution should be exercised given the risk of contrast peritonitis if the PEG is in communication with the peritoneal cavity, water soluble contrast are therefore preferred [9]. In our case, the suspicion of the diagnosis was entertained by the initial CT scan done in the ED and confirmation made with an upper endoscopy demonstrating fecal material entering the stomach through a fistula and colonoscopy showing a PEG tube with internal bumper in the transverse colon, which could easily be mobilized through the colocutaneous fistula.

The management of an iatrogenic fistula resulting from a PEG tube would depend on the presence or absence of peritonitis, with the latter being managed successfully through a nonsurgical approach [12]. A conservative approach can be pursued with the drawback that it would take days or weeks for the fistula to heal, resulting in delays with reintroduction of feeding and increased hospital length of stay [13]. Attempts at endoscopic repair of the iatrogenic fistula resulting from a malpositioned PEG tube have been reported in the literature. Nici et al. reported closure of the gastrocolic fistula in a patient with partial colectomy for acute diverticulitis, using the scope resolution clips aided by fluoroscopy [14]. Our patient had repair of the 10 mm gastrocolic fistula with an over-the-scope padlock clip (US Endoscopy), while the colocutaneous fistula was closed with resolution clips (Boston Scientific). The colocutaneous fistula could not be repaired with an OTSC as the location in the transverse colon could not be reached with a gastroscope, and this was subsequently repaired with through the scope clips using a pediatric colonoscope. There are also limits to repair of these fistulas that can be extrapolated from a study by Matthes et al. They showed in animal studies that a single OTSC can be used to close defects in the stomach ranging from 5 to 20 mm and from 10 to 30 mm in the colon [15]. Other nonsurgical modalities that can be used to repair these fistulas include but not limited to endoloops, sutures, stents, injectables, and coagulants.

Closure of both fistulas was confirmed by air insufflation, and the patient tolerated the procedures with no further complications. This nonsurgical endoscopic repair enabled resumption of enteral feeding through the nasogastric tube 48 hours after procedure. The rest of his hospital course was uneventful, and 10 days later, he had another PEG tube placed at a different location. He was subsequently discharged back to a long-term care facility with no further concerns with the feeding tube during a seven-month follow-up.

## 4. Conclusion

With increasing use of the PEG tube for nutritional support in clinical setting, care should be taken to adhere to strict aseptic measures and other risk mitigating strategies that would help to reduce the chance of complications. Some of the encountered complications include but not limited to bleeding, infection, and iatrogenic fistula like a gastrocolic and colocutaneous as was the case in our index patient. The latter occurring especially when care is not taken to ensure that the colon is not juxtaposed between the stomach and abdominal wall. Nonsurgical repair is an option in patients without peritonitis and can be achieved with the use of over-the-scope and through-the-scope clips, aiding timely return to enteral feeding and subsequent discharge to home or to long-term care facilities.

## References

[1] Taheri M. R., Singh H., Duerksen D. R. (2011). Peritonitis after gastrostomy tube replacement. *Journal of Parenteral and Enteral Nutrition*.

[2] Dwyer K. M., Watts D. D., Thurber J. S., Benoit R. S., Fakhry S. M. (2002). Percutaneous endoscopic gastrostomy: the preferred method of elective feeding tube placement in trauma patients. *Journal of Trauma and Acute Care Surgery*.

[3] Schrag S. P., Sharma R., Jaik N. P. (2007). Complications related to percutaneous endoscopic gastrostomy (PEG) tubes. A comprehensive clinical review. *Journal of Gastrointestinal and Liver Diseases*.

[4] Mamel J. J. (1989). Percutaneous endoscopic gastrostomy. *The American Journal of Gastroenterology*.

[5] Gauderer M. W. L. (2001). Percutaneous endoscopic gastrostomy-20 years later: a historical perspective. *Journal of Pediatric Surgery*.

[6] McClave S. A., Neff R. L. (2006). Care and long-term maintenance of percutaneous endoscopic gastrostomy tubes. *Journal of Parenteral and Enteral Nutrition*.

[7] Sasaki T., Fukumori D., Sakai K., Sato M., Ohmori H., Yamamoto F. (2004). The safety and feasibility of percutaneous endoscopic gastrostomy placement. *Hepatogastroenterology*.

[8] Kinoshita Y., Udagawa H., Kajiyama Y. (1999). Cologastric fistula and colonic perforation as a complication of percutaneous endoscopic gastrostomy. *Surgical Laparoscopy, Endoscopy & Percutaneous Techniques*.

[9] Lohiya G.-S., Tan-Figueroa L., Krishna V. (2010). Intermittent diarrhea as a delayed presentation of percutaneous endoscopic gastrostomy (PEG)-associated fistula. *The Journal of the American Board of Family Medicine*.

[10] Friedmann R., Feldman H., Sonnenblick M. (2007). Misplacement of percutaneously inserted gastrostomy tube into the colon: report of 6 cases and review of the literature. *Journal of Parenteral and Enteral Nutrition*.

[11] Guloglu R., Taviloglu K., Alimoglu O. (2003). Colon injury following percutaneous endoscopic gastrostomy tube insertion. *Journal of Laparoendoscopic & Advanced Surgical Techniques*.

[12] Kilmartin C., Brotzman G. L., Regan P. (1996). Colocutaneous fistula as a complication of PEG tube placement. *The Journal of Family Practice*.

[13] Kim H. S., Lee D. K., Baik S. K., Kwon S. O. (2002). Endoscopic management of colocutaneous fistula after percutaneous endoscopic gastrostomy. *Endoscopy*.

[14] Nici A., Hussain S., Rubin M., Kim S. (2013). Repair of a gastrocolic fistula using a wire-guided, simultaneous dual scope approach. *Endoscopy*.

[15] Matthes K., Jung Y., Kato M., Gromski M. A., Chuttani R. (2011). Efficacy of full-thickness GI perforation closure with a novel over-the-scope clip application device: an animal study. *Gastrointestinal Endoscopy*.

